# Whole-genome microarray analysis and functional characterization reveal distinct gene expression profiles and patterns in two mouse models of ileal inflammation

**DOI:** 10.1186/1471-2164-13-377

**Published:** 2012-08-06

**Authors:** Leela Rani Avula, Dries Knapen, Roeland Buckinx, Lucia Vergauwen, Dirk Adriaensen, Luc Van Nassauw, Jean-Pierre Timmermans

**Affiliations:** 1 Department of Veterinary Sciences, Laboratory of Cell Biology and Histology, University of Antwerp, Groenenborgerlaan 171, Antwerp, B-2020, Belgium; 2 Department of Biology, Laboratory of Ecophysiology, Biochemistry and Toxicology, University of Antwerp, Groenenborgerlaan 171, Antwerp, B-2020, Belgium; 3 Faculty of Medicine and Health Sciences, Laboratory of Human Anatomy and Embryology, University of Antwerp, Groenenborgerlaan 171, Antwerp, B-2020, Belgium; 4 Department of Veterinary Sciences, Laboratory of Physiology and Biochemistry of Domestic Animals, University of Antwerp, Universiteitsplein 1, Antwerp, B-2610, Belgium

**Keywords:** Gene expression, Intestinal inflammation, Ileum, Murine models, Intestinal schistosomiasis, TNBS-induced ileitis, Whole-genome microarrays, Pathways, Over-representation analysis

## Abstract

**Background:**

Although a number of intestinal inflammatory conditions pertain to the ileum, whole-genome gene expression analyses in animal models of ileal inflammation are lacking to date. Therefore, we aimed to identify and characterize alterations in gene expression in the acutely inflamed ileum of two murine models of intestinal inflammation, namely intestinal schistosomiasis and TNBS-induced ileitis, compared to healthy controls. To this end, we used whole-genome microarrays, followed by bioinformatics analyses to detect over-represented Kyoto Encyclopedia of Genes and Genomes pathways and Gene Ontology categories.

**Results:**

Following screening of almost all known mouse genes and transcripts represented on the array, intestinal schistosomiasis and TNBS-induced ileitis yielded 207 and 1417 differentially expressed genes, respectively, with only 30 overlapping concordantly changed genes. Functional category groups consisting of complement and coagulation cascades, extracellular matrix (ECM)-receptor interaction, Fc epsilon receptor I signaling pathways and protein activation cascade, cell adhesion categories were over-represented in the differential gene list of intestinal schistosomiasis. Antigen processing and presentation, cell adhesion molecules, ABC transporters, Toll-like receptor signaling pathways and response to chemical stimulus categories were over-represented in the differential gene list of TNBS-induced ileitis. Although cytokine-cytokine receptor interaction, intestinal immune network for IgA production, focal adhesion pathways and immune, inflammatory and defense response categories were over-represented in the differential gene lists of both inflammation models, the vast majority of the associated genes and changes were unique to each model.

**Conclusions:**

This study characterized two models of ileal inflammation at a whole-genome level and outlined distinct gene expression profiles and patterns in the two models. The results indicate that intestinal schistosomiasis involves Th2 responses, complement activation, protein activation and enhanced ECM turnover, while TNBS-induced ileitis involves Th17 responses, defective antigen processing and presentation and altered Toll-like receptor-mediated responses. Signs of an impaired epithelial barrier are apparent in both inflammation models. Furthermore, the comprehensive differential gene list and functional groups provided by this study constitute an interesting starting point to explore new targets and extended functional networks dealing with small bowel inflammation.

## Background

Studies in humans and animal models of intestinal inflammation have greatly improved our understanding of some of the underlying dysregulated immune mechanisms, which in turn has promoted the development of new therapeutic strategies. Some of these studies, which were based on whole-genome microarrays for the identification of altered gene expression and transcript regulation at the whole-genome level, have been instrumental in characterizing inflammatory conditions at the molecular genetic level, in obtaining a global picture of the underlying functional processes, as well as in identifying many of the key molecules involved. However, to date, most of these studies involving whole-genome gene expression assays in humans and in animal models of intestinal inflammation have generally targeted the large intestine rather than the small intestine, simply because of tissue accessibility and because of the fact that the majority of the frequently used animal models of both acute and chronic intestinal inflammation involve the large intestine rather than the small intestine [1-5]. As a result, only little is known about the molecular genetic changes underlying small bowel inflammation at the whole-genome level [6]. Nonetheless, it is widely known that a number of major intestinal inflammatory conditions pertain to the ileum, which contains the most complex part of the gut-associated lymphoid tissue, the Peyer's patches, and also harbors a very high luminal concentration of microorganisms [7]. For instance, human Crohn's disease (CD) is most frequently localized in the terminal ileum [7], and it also becomes increasingly evident that human ulcerative colitis (UC) might also involve the terminal ileum due to further extension of the inflammatory process either by backwash from the colon or by other factors [8,9]. A few murine models of small bowel inflammation bearing resemblance to CD have provided vital information on some of the mechanisms underlying small bowel inflammation, but, to the best of our knowledge, whole-genome gene expression studies in the full-thickness ileum have been performed neither in these animal models, nor in other animal models involving any other type of small bowel inflammation to date [10-13].

Studies in mouse revealed that gene expression patterns vary between the anatomically defined regions of the gastrointestinal (GI) tract, such as the stomach, small intestine and large intestine [14]. Moreover, a study based on gene expression in humans suggests that immune regulation is different in ileal and colonic inflammation, which might have important consequences for therapeutic intervention [15]. Consequently, gene expression data on large bowel inflammation cannot be completely extrapolated to other regions of the GI tract, and as such a detailed analysis of the genes involved in small intestinal inflammation is highly desirable. These findings, along with the above-mentioned lack of whole-genome gene expression studies in full-thickness ileum in animal models of intestinal inflammation, stress the urgent need to elaborate on the molecular genetic changes underlying ileal inflammation in view of developing better therapeutic strategies. Therefore, the objective of this whole-genome microarray study was to identify and characterize gene expression changes in the acutely inflamed ileum of two diverse murine models of intestinal inflammation, namely, *Schistosoma mansoni*-induced intestinal schistosomiasis, which is a non-inflammatory bowel disease model and 2,4,6-trinitrobenzene sulfonate (TNBS)-induced ileitis, which is an inflammatory bowel disease (IBD) model, compared to healthy controls.

Schistosomiasis is a highly prevalent tropical disease caused by the blood-dwelling trematode of the genus *Schistosoma*, and, although successfully controlled in many countries, remains a major public health problem with an estimated 200 million infected people worldwide [16]. After infection by larvae of *S. mansoni*, adult worms reach and reproduce in the mesenteric vessel bed and parasite eggs get entrapped in the portal and mesenteric venous systems. A proportion of these eggs penetrate through the liver and intestinal wall to enter the lumen, thereby generating a granulomatous inflammation and subsequent fibrosis [17-19]. Intestinal schistosomiasis has been widely studied in the context of granulomatous inflammation. Moreover, *Schistosoma*-derived eggs and soluble worm proteins have been shown to alleviate intestinal inflammation in animal models of IBD [20,21]. Animal models of granulomatous inflammation, such as schistosomiasis, permit a detailed analysis of basic immune mechanisms involved in chronic immunoregulation that is also relevant to other species [22,23]. Therefore, further study of murine intestinal schistosomiasis at the molecular genetic level will lead to a better understanding of its immunomodulatory and immunoregulatory mechanisms.

TNBS is a haptenating agent that immunogenizes autologous or microbiota proteins in the mucosa after disruption of the intestinal epithelial barrier by ethanol. Intraluminal instillation of TNBS in ethanol is known to generate classical models of intestinal inflammation mimicking some aspects of CD [24]. TNBS is one of the best-known chemical agents used for the induction of colitis and ileitis in animal models [25-27]. TNBS-induced inflammation has been widely studied for its resemblance to CD and is considered an apt model to study the acute local inflammatory response as well as the subsequent established and delayed-type hypersensitivity reactions [26].

The present study reports on altered whole-genome gene expression and the associated functional categories and pathways underlying acute ileal inflammation in mouse induced by the two aforementioned etiologic factors, i.e., *S. mansoni* parasite-derived eggs, and a chemical agent, TNBS. Our findings collectively provide a solid basis and a starting point for a better understanding of a number of crucial and novel functional processes underlying ileal inflammation initiated by different causative agents.

## Methods

### Animal handling

All experiments were approved by the ethics committee of the University of Antwerp. Adult female C57BL/6 J mice, purchased from JANVIER (Le Genest St Isle, France), were given a standard pellet diet plus water ad libitum, and were housed in a 12 h/12 h light/dark cycle at constant temperature (22°C). The animals were divided into three groups: a healthy control group and two inflamed groups, i.e., a group with *S. mansoni*-induced intestinal schistosomiasis and a group with TNBS-induced ileitis (n = 3 in each group).

Mice were infected with *S. mansoni* according to the method of Yolles et al. [28]. Briefly, the mice were anesthetized with an intraperitoneal injection of sodium pentobarbital (60 mg kg^-1^; NEMBUTAL; Sanofi, Brussels, Belgium) followed by an intraperitoneal injection of 1 ml of sterile water containing 130 freshly shed cercariae of a Puerto Rico strain of *S. mansoni*. The cycle of *S. mansoni* was maintained by passage through *Biomphalaria glabrata* snails.

TNBS-ileitis was induced by laparotomy according to a modified procedure of Pontell et al. [29,30]. Briefly, after fasting for 24 h, mice were anesthetized using a mix of medetomidine hydrochloride (0.5 mg kg^-1^; DOMITOR; Pfizer, New York, NY, USA) and ketamine hydrochloride (50 mg kg^-1^; ANESKETIN; Eurovet, Bladel, the Netherlands) dissolved in physiological solution and administered intraperitoneally. After having been shaved and disinfected, the lower abdomen was incised and the ileum was exteriorized on sterile gauze. A volume of 0.1 ml of 25 mg ml^-1^ TNBS (Sigma-Aldrich, St. Louis, MO, USA) dissolved in 25% ethanol was injected transmurally into the lumen of the ileum approximately 2 cm proximal to the ileo-cecal junction. The ethanol-carrier is included in the basic protocol and is an essential part of the TNBS-inflammation model since it compromises the epithelial barrier, allowing the access of hapten and luminal contents to subepithelial immune cells, thus initiating the immune response [26]. The laparotomy was sutured in two layers using non-resorbable sutures. Before closing the midline incision, a solution containing marbofloxacine (2 mg kg^-1^; MARBOCYL; Vetoquino S.A., Lure cedex, France) was injected into the peritoneal cavity. After surgery, animals were maintained in a controlled environment for 24 h.

*S. mansoni*-infected animals were sacrificed 8 weeks post infection at the acute stage of intestinal schistosomiasis. TNBS-treated animals were sacrificed 24 h post induction at the acute stage of ileitis. All control and inflamed animals were age-matched at the time of tissue retrieval. Since the handling procedures used to induce inflammation in our study are established methods that are not known to induce any inflammatory changes per se, and also taking the high cost of microarray experiments, as well as the reduction of the number of animals for ethical reasons into consideration, we did not consider it necessary to include any additional controls for the inflammation induction procedures in our microarray study [29,30]. Although the impact of the survival surgery protocol in the TNBS-treated group was not controlled for in the healthy control group in our study, other studies using the same established surgery protocol and sham treatment revealed a minimal impact of the procedure in itself on these animals [25,27,29,30]. Therefore, we estimate that the gene-expression data in our study might have had a minimal chance of derivation from the surgery protocol by itself. All animals were euthanized in the morning by cervical dislocation, followed by exsanguination. Full-thickness terminal ileum was dissected out and rinsed with Krebs solution (117 mM NaCl, 5 mM KCl, 2.5 mM CaCl_2_·2H_2_O, 1.2 mM MgSO_4_·7H_2_O, 25 mM NaHCO_3_, 1.2 mM NaH_2_PO_4_·2H_2_O and 10 mM glucose; pH 7.4). Pieces of ileum weighing around 100 mg each were snap-frozen in liquid nitrogen and stored at −80°C for RNA extraction. A small part of the ileum taken from the same anatomical location as the samples used for the microarray experiments, was fixed for 2 h at room temperature in 4% paraformaldehyde in 0.1 M phosphate buffer (pH 7.4) and processed for paraffin embedding. Five μm-thick paraffin sections were stained with hematoxylin-eosin to verify the degree of inflammation. Two pathologists blinded to the disease status and experimental results evaluated the histological specimens by light microscopy.

### RNA extraction

Total RNA was isolated using the TRIzol® extraction method (Life Technologies, Merelbeke, Belgium). In brief, tissues were ground to a fine powder with mortar and pestle in liquid nitrogen, and the homogenized tissues mixed with TRIzol® were used for total RNA isolation according to the manufacturer's protocol. RNA extracts were purified using DNAse I and RNAse inhibitor (Fermentas, St. Leon-Rot, Germany) followed by phenol–chloroform extraction [31]. RNA integrity and purity were verified by denaturing formaldehyde-agarose gel electrophoresis and measurement of 260/230 nm and 260/280 nm absorbance ratios by ND-1000 spectrophotometry (NanoDrop Technologies, Rockland, DE, USA). RNA samples were used only if the ribosomal bands showed no degradation on the gel and the 260/280 and 260/230 absorbance ratios were between 1.7 and 2.1. A total of 10 ileal total RNA samples were prepared (3 control samples, 3 *S. mansoni*-infected samples, 3 TNBS-treated samples and one reference sample containing a pool of equimolar amounts of all control total RNA samples).

### Fluorescent cRNA labeling and oligonucleotide microarrays

The two-colour microarray-based gene expression analysis protocol (version 5.7, http://www.agilent.com) was followed to construct and fluorescently label cRNA from total RNA extracts. Briefly, probes were prepared by converting an aliquot of 1 μg total RNA from each sample into labeled cRNA, using reagents from the two-colour spike-in kit (catalogue No. 5188–5279, Agilent Technologies, Diegem, Belgium) and a two-colour Quick amp labeling kit (catalogue No.5190-0444, Agilent Technologies). Total RNA was reverse transcribed into first- and second-strand cDNA, after which first-strand cRNA was constructed using the second-strand cDNA as a template, in the presence of either Cy3-CTP or Cy5-CTP. Using this method the target material was simultaneously amplified at least a hundred times. The labeled cRNA probes were purified to ensure removal of unbound Cy dyes using the RNeasy mini spin column kit (catalogue No.74104, Qiagen Benelux B.V., Venlo, the Netherlands). Labeling efficiency was determined at 550 nm (Cy3) and 650 nm (Cy5) by ND-1000 spectrophotometry (NanoDrop Technologies).

A custom whole-genome gene expression high-definition oligo microarray platform, (design ID 021695, catalogue No. G2514F), was created and made publicly available in the Gene Expression Omnibus (GEO) database (GPL14111). This custom microarray 4X44K platform represented almost all known genes in the mouse genome and their resulting transcripts in over 41000 60-mer oligonucleotide probe features. Experiments were performed on this platform using a reference design as indicated by Knapen et al. [32]. This design consisted of a total of 11 arrays for three biological replicates of control, *S. mansoni*-infected and TNBS-treated tissue samples as well as two dye flipped technical replicates of a control and a *S*. *mansoni*-infected tissue sample (see Gene Expression Omnibus (GEO) series accession number GSE31265). Using the Gene expression hybridization kit (catalogue No. 5188–5242, Agilent Technologies) a hybridization sample volume of 100 μl was applied on each array containing a total of 825 ng of test sample hybridized against the complementarily labeled reference cRNA. Subsequently, the microarray slides were assembled in the slide chamber and incubated at 65°C for 17 h in a rotating hybridization chamber (Genetix Ltd, New Milton, Hampshire, UK). Slides were stringently washed with wash buffers and acetonitrile and finally submersed in stabilization and drying solution (Agilent Technologies) to prevent ozone-induced Cy5 degradation. Subsequently, the slides were placed in a nitrogen purge box for temporary storage and scanned immediately.

### Microarray analysis

Slides were scanned in the Genepix Personal confocal 4100A scanner (Axon Instruments, Union City, CA, USA) at a resolution of 5 μm and at a wavelength close to 532 nm and 635 nm for Cy3 and Cy5 channels, respectively. The photomultiplier tube voltages were adjusted to obtain an overall green/red ratio of one. Images were processed using the GenePix Pro 4.1 software (Axon Instruments) for the identification and quantification of the fluorescent signal intensities of the spots [31]. The resulting files were saved as Genepix results (GPR) files, and were imported into the BioArray Software Environment database (BASE 1.2.12, accessible at http://www.islab.ua.ac.be/base/), a free MIAME platform-based microarray analysis method customized by the Intelligent Systems Laboratory (University of Antwerp, Antwerp, Belgium). Through this database, statistical analysis was performed using the R package limma (linear models for microarray data). Limma is a moderated *T*-statistic approach that detects differentially expressed genes between test groups, given the natural variance within these test groups, adjusted for multiple testing [31,33-35]. Spots for which red or green FG < BG + 2SD as well as spots that were flagged due to artifacts received a weight of zero before analysis (FG: median foreground intensity, BG: average local background intensity calculated over the full microarray, SD: standard deviation of local background intensities) [36]. The median background signal was subtracted from the median foreground signal. The median intensity data were corrected for background using a normal-exponential convolution model with method “normexp”, offset = 50 using the function backgroundCorrect, and loess-normalized using the function normalizeWithinArrays, after which the data were normalized for between-array normalization using vsn (variance stabilization) [31,37,38]. The two inflammation models were contrasted against the controls, linear models were fitted to intensity ratios, after which probes were ranked in order of evidence of differential expression using an empirical Bayes method [33]. Probes were considered differentially expressed when the p-value was < 0.05, adjusted p-value (calculated for multiple testing corrections (MTC) on the obtained p-values by the Benjamini and Hochberg false discovery rate method) was < 0.1 and log_2_FC was > 1 or < −1 (log_2_FC: log_2_ fold change), resulting in a list of differentially expressed probes for each inflammation model. The microarray data have been deposited in the National Center for Biotechnology Information GEO (NCBI GEO; http://www.ncbi.nlm.nih.gov/geo) and are accessible under the GEO series accession number GSE31265.

### Bioinformatics data mining and over-representation analysis

Growing evidence that certain sets of genes acting in concert contribute to the etiology of disease conditions has led to an increased interest in identifying networks or groups of genes, rather than single genes. Moreover, often subtle changes in gene expression, or sometimes the lack of such changes, can equally contribute significantly to changes in function, and these biologically meaningful changes sometimes go undetected by single-gene analysis [39,40]. Therefore, we set out to identify biologically relevant category groups in the two inflammation models of our study by performing a function-based characterization of the differentially expressed genes. To this end, using the stand-alone version of the GO-Elite tool (http://www.genmapp.org/go_elite) [41], we performed a pathway or functional over-representation analysis of the differentially expressed genes for Kyoto Encyclopedia for Genes and Genomes (KEGG) pathways and GO categories. We chose the KEGG pathway analysis rather than analysis of other pathway databases since KEGG is considered to be one of the biggest available pathway databases compiled from several literature sources, especially for mouse [42,43]. Since the stand-alone version of GO-Elite does not work for an over-representation analysis of KEGG pathways, we first downloaded the KEGG gpml archive from the PathVisio website (http://www.pathvisio.org), replaced other pathway flat-file relationships already existing in GO-Elite with that of KEGG, and then were able to perform a functional analysis of KEGG pathways in GO-Elite. The input gene set for our over-representation analysis consisted of the differential gene list corresponding to either of the two inflammation models, while the whole genome was used as the denominator or reference background. We used the GO-elite tool with the default options: significance threshold of z-score > 2 and at least three genes from the input list in the enriched KEGG pathway or GO category. GO-elite generated a full list of KEGG and GO annotations associated with all differentially expressed genes, calculated over-representation statistics and provided a minimal list of relevant pruned annotations (filtered for highest scoring terms along each division or branch of the hierarchy and non redundancy in enriched terms) for each inflammation model. The pruned list included main statistics, such as z-score > 2 (calculated using the expected value and standard deviation of the number of genes meeting the criterion on a pathway under a hypergeometric distribution), permutation p-value (calculated by selecting the original number of input identifiers at random from the denominator identifiers 2000 times), adjusted permutation p-value (calculated using the Benjamini-Hochberg method), average log_2_FC (for each enriched term based on associated differential genes) and average adjusted p-value (for each enriched term based on associated differential genes) for the KEGG and GO terms [41,44]. From this pruned list, we finally set three strict threshold criteria for significance in our over-representation analysis, i.e., a z-score > 2, at least three genes from the input list in the KEGG pathway or GO category and an adjusted permutation p-value ≤ 0.35 [41,44]. These criteria enabled us to identify highly relevant category groups (KEGG pathways + GO biological process categories) in the two inflammation models of our study. Further, GenMAPP (http://www.genmapp.org/) was used for visualization of the gene expression data in the over-represented KEGG pathways, [41-45].

In addition to the above-mentioned analyses, we briefly compared the list of differentially expressed genes in the two inflammation models of our study with the list of genes previously known to be associated with IBD to assess the degree of similarity between our inflammation models and IBD.

## Results and discussion

The data presented in this study are derived from the analysis of full-thickness murine ileal tissue during physiological and pathophysiological states – i.e., healthy control and the acute stages of intestinal schistosomiasis and TNBS-induced ileitis, respectively, which allowed us to characterize and compare the two models in an intact heterogeneous ileal tissue system. Whole-genome microarrays containing almost all known mouse genes with their resulting transcripts were screened for differential expression in order to unequivocally disclose the differentially expressed genes in the two models and to identify the key functions involved, in terms of KEGG pathways and biological processes.

### Histology-hematoxylin-eosin stainings

**Figure 1:**
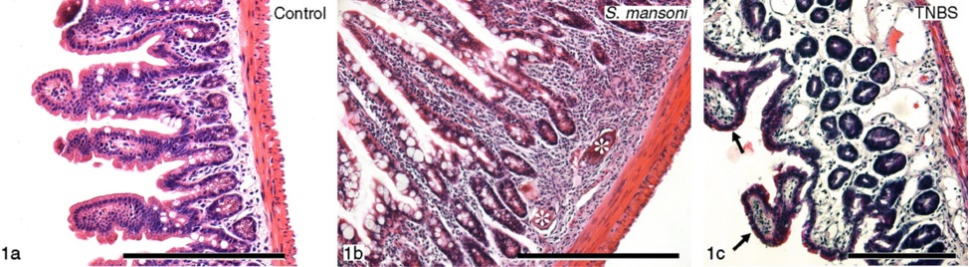
**Hematoxylin-eosin-stained paraffin sections of control and inflamed ileum. a:** The control ileum showed no pathological signs of inflammatory activity **b:** The *S. mansoni*-infected ileum was characterized by the presence of granulomas with entrapped parasite eggs (asterisk), thickening of the tunica muscularis and broadening of the villi **c:** The TNBS-treated ileum showed ablated and broken villi, signs of regeneration of the mucosal epithelium (arrows), subepithelial cysts and a high number of immune cells in the lamina propria as well as in the vicinity of enteric ganglia. Scale bar = 200 μm.

No pathological signs of inflammatory activity were observed in the ileum of control mice (Figure 1a), while the ileum of *S. mansoni*-infected mice was characterized by the presence of granulomas consisting of macrophages, eosinophilic granulocytes and lymphocytes surrounding the entrapped parasite eggs in the submucosa and mucosa, by thickening of the tunica muscularis and by broadening of the intestinal villi (Figure 1b). The diffuse mucosal inflammation consisted of granulocytes and scarce lymphocytes. These observations were consistent with previous descriptions [17]. Observations in the ileum of TNBS-treated mice were similar to those described in guinea-pig [30]. The villi showed a destructed structure and were ablated, resulting in debris in the lumen, although signs of restoration and regeneration of the mucosal epithelium were observed. Subepithelial cysts and a high number of infiltrated granulocytes, macrophages, along with some lymphocytes within the lamina propria or closely associated with enteric ganglia, were also seen (Figure 1c). As such, all inflamed animals showed obvious acute inflammatory changes, consistent with previous literature data, at the time of tissue retrieval.

As ethanol was used as a vehicle for inducing inflammation in the TNBS-ileitis model, to control for changes induced by ethanol toxicity in itself, the ileum of mice treated with 25% ethanol alone was also examined by hematoxylin-eosin stainings. Observations in these mice showed no histopathological changes in the ileum after 24 h (data not shown). These findings are consistent with other studies in mice [27].

### Microarray analysis and gene expression profiling

Statistical limma analysis indicated that 236 and 1685 probes showed differential expression in the *S. mansoni*-infected and the TNBS-treated ileum, respectively (log_2_FC > 1 or < −1; p-value < 0.05, adjusted p-value < 0.1, MTC Benjamini-Hochberg). After removal of duplicates, this differentially expressed probe list was found to correspond to a total of 207 unique genes (172 upregulated and 35 downregulated) in the *S. mansoni*-infected ileum, and to a total of 1417 unique genes (611 upregulated and 806 downregulated) in the TNBS-treated ileum. Analysis of shared gene expression profiles revealed that 48 genes shared differential expression between the two models, i.e., 20 commonly shared upregulated genes, 10 commonly shared downregulated genes and 18 genes that showed opposite changes in the two inflammation models (17 genes upregulated in the *S. mansoni*-infected ileum and downregulated in the TNBS-treated ileum, and 1 gene downregulated in the *S. mansoni*-infected ileum and upregulated in the TNBS-treated ileum). Detailed information on all differentially expressed genes is found in the additional files (see Additional file 1 and Additional file 2).

The above findings clearly demonstrate that, in general, the number of genes showing differential expression is higher in the TNBS-treated ileum than in the *S. mansoni*-infected ileum (approximately 6 times more), implying that TNBS-induced ileitis has a more profound impact on ileal gene expression than intestinal schistosomiasis. Furthermore, the vast majority of differentially expressed genes in the *S. mansoni*-infected ileum were upregulated, while downregulated genes narrowly outnumbered upregulated genes in the TNBS-treated ileum. More interestingly, only a minority (30 genes) of all the differential genes overlapped between the *S. mansoni*-infected ileum and the TNBS-treated ileum in a concordant manner. Together, these findings indicate that the gene expression profiles in the two inflammation models are considerably different, likely owing to the different inflammation-inducing agents.

### Gene ontology (GO) and pathway over-representation analysis

The full list of KEGG and GO annotations in GO-Elite showed that of the whole mouse genome represented on the arrays, 5699 individual genes were contained within the GO-elite KEGG database and 17730 individual genes were contained within the GO-elite GO database, and were used for analysis. Of the 207 unique genes that were differentially expressed in the *S. mansoni*-infected ileum, 42 genes could be linked to a KEGG pathway term and 144 genes to a GO term. Of the 1417 unique genes that were differentially expressed in the TNBS-treated ileum, 232 genes could be linked to a KEGG pathway term and 1050 genes to a GO term. Additional files 3 and 4 detail all the differentially expressed genes that could be linked to a KEGG pathway and/or GO term in the *S. mansoni*-infected and the TNBS-treated ileum respectively (see Additional files 3 and 4).

The above findings clearly show that although the vast majority of the differentially expressed genes in the two inflammation models of our study could be linked to a GO term and/or a KEGG pathway term, there still remained a significant number of differentially expressed genes that could not be associated with a KEGG pathway term (≈80% in the *S. mansoni*-infected ileum and ≈ 84% in the TNBS-treated ileum) or a GO term (≈30% in the *S. mansoni*-infected ileum and ≈ 26% in the TNBS-treated ileum).

Based on the above-described threshold criteria for over-representation analysis described in the methods section, nine KEGG pathways were over-represented in the upregulated gene list of the *S. mansoni*-infected ileum. Only one KEGG pathway was over-represented in the downregulated gene list of the *S. mansoni*-infected ileum. Twenty-six GO biological process categories were over-represented in the upregulated gene list of the *S. mansoni*-infected ileum, and three GO biological process categories were over-represented in the downregulated gene list of the *S. mansoni*-infected ileum. Detailed information on all over-represented KEGG pathways and GO categories in the *S. mansoni*-infected ileum are found in additional file 5 (see Additional file 5).

Twenty-six KEGG pathways were over-represented in the upregulated gene list of the TNBS-treated ileum. Twenty-eight KEGG pathways were over-represented in the downregulated gene list of the TNBS-treated ileum. Ninety-seven GO biological process categories were over-represented in the upregulated gene list of the TNBS-treated ileum and seventy-one GO biological process categories were over-represented in the downregulated gene list of the TNBS-treated ileum. Detailed information on all over-represented KEGG pathways and GO categories in the TNBS-treated ileum are found in additional file 6 (see Additional file 6).

### Identification of functionally meaningful category groups

Given the high number of over-represented KEGG and GO terms, we considered it relevant to make a shortlist of the most significant category groups. Based on the high overlap of individual genes observed among some of the high scoring KEGG pathways and GO categories, five functionally meaningful category groups (KEGG pathways + GO categories) were finally obtained in the *S. mansoni*-infected ileum (Additional file 7: Table S1):

1. Cytokine-cytokine receptor interaction pathway and immune and defense response categories;

2. Intestinal immune network for immunoglobulin A (IgA) production pathway and immune response category;

3. Complement and coagulation cascades pathway and protein activation cascade category;

4. Extracellular matrix (ECM)-receptor interaction pathway and cell adhesion category;

5. Fc epsilon RI (FcεRI) signaling pathway and regulation of acute inflammatory response category.

Six functionally meaningful category groups were finally obtained in the TNBS-treated ileum (Additional file 8: Table S2):

1. Cytokine-cytokine receptor interaction pathway and inflammatory and immune response categories;

2. Intestinal immune network for IgA production pathway and immune response category;

3. Antigen processing and presentation pathway and antigen processing and presentation of endogenous antigen, of exogenous peptide antigen and of peptide or polysaccharide antigen via major histocompatibility complex (MHC) class II categories;

4. Cell adhesion molecules pathway and cell adhesion category;

5. Toll-like receptor (TLR) signaling pathway and immune and inflammatory response categories;

6. ABC transporters pathway and response to chemical stimulus category.

These functional category groups will be elaborated on below. The category groups pertaining to both inflammation models will be discussed first, followed by those that are relevant only to the *S. mansoni*-infected ileum and then those relevant only to the TNBS-treated ileum. Wherever necessary, in order to group important information, changes pertaining to genes that are not contained in the category group but are of relevance will also be discussed.

### Cytokine-cytokine receptor interaction pathway and immune response category group

**Figure 2:**
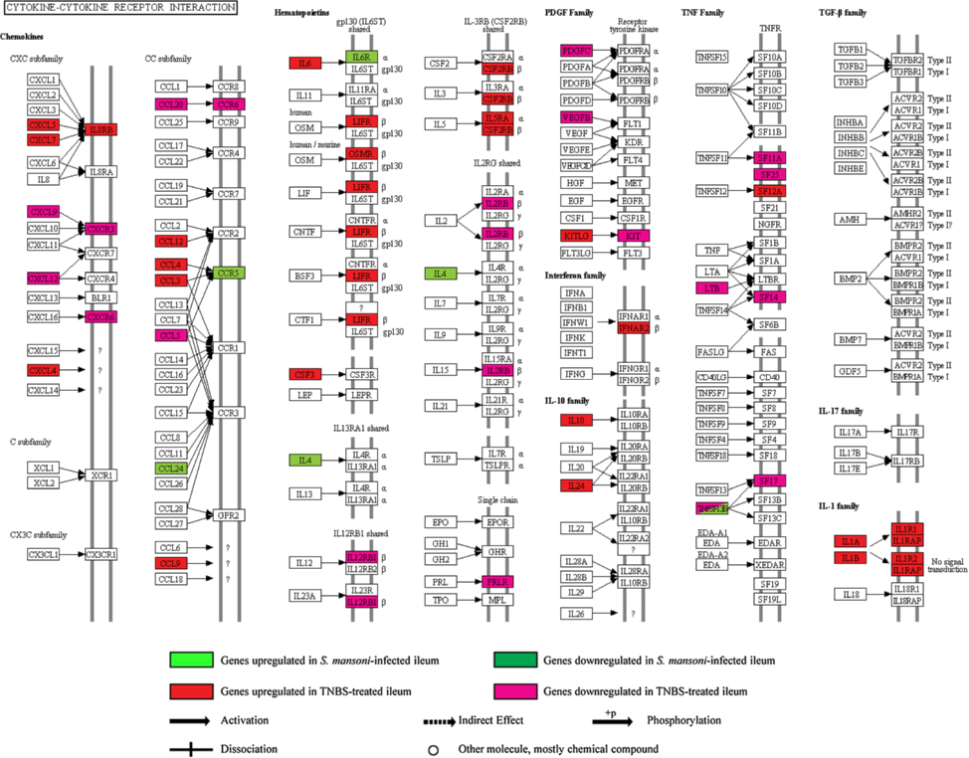
**Illustration of the KEGG cytokine-cytokine receptor interaction pathway with location of 6 and 18 associated genes that were differentially expressed in the *S. mansoni*-infected and the TNBS-treated ileum, respectively.**

(Additional file 1, Additional file 2, Additional file 7: Table S1 and Additional file 8: Table S2) (Figure 2).

In the *S. mansoni*-infected ileum, our findings suggest a predominant presence of polarized Th2 responses during acute intestinal schistosomiasis, and that the cytokine-cytokine receptor interaction pathway-immune (and defense) response category group is upregulated. These findings are in agreement with previous reports showing that the inflammatory reaction to schistosome eggs in the mouse intestine is predominantly a Th2-type response [46]. The cytokine-cytokine receptor interaction pathway and immune (and defense) response category group shared 6 genes (*Ccl24*, *Ccr5*, *Il18r1*, *Il4*, *Il6ra*, *Tnfsf13b*), all of which showed increased expression. Increased expression of *Il4*, *Ccl24* and *Ccr5* is indicative of Th2-type responses [47-49] and IL4 is a hallmark cytokine for Th2-type T cell responses [47]. Upregulation of *Ccl24* (eotaxin-2) is associated with recruitment of immune cells including eosinophils and Th2 cells in the GI mucosa during helminth infection [48]. The increased expression of the *Ccr5* found in our study lends further support to literature data showing that CCR5 plays a role in limiting the granulomatous and Th2 responses, thereby reducing severity of experimental schistosomiasis [49]. Furthermore, the increased expression of resistin-like beta (*Retnlb*/*Fizz2*) in the *S. mansoni*-infected ileum supports the notion that the protein encoded by this gene, intestinal goblet cell-derived RELM-beta/FIZZ2, is a Th2 cytokine-induced immune-effector molecule produced in resistance to *S. mansoni* infection [50]. RELM-beta/FIZZ2 has also been associated with the initiation of ileitis in animal models of CD [10]. The highly increased expression of other Th2 cytokine-associated genes, such as chitinase-3-like genes *Chi3l3* and *Chi3l4* indicate their probable involvement in host/microbial interactions and thereby in the disease pathogenesis in this model [51].

In the TNBS-treated ileum, our findings suggest that proinflammatory responses, Th17 priming and regulatory mechanisms are enhanced during acute TNBS-induced ileitis, and that the cytokine-cytokine receptor interaction pathway-immune (and inflammatory) response category group is upregulated under this condition. This particular category group shared 8 genes (*Ccl3*, *Ccl4*, *Ccl9*, *Cxcl5*, *Il1b*, *Il6*, *Pf4*, *Ppbp*), all of which showed increased expression. Increased expression of *Il6* and *Il1b* and of genes such as the transcription factor *Stat3*, in the TNBS-treated ileum indicate changes that are associated with Th17 cell induction and differentiation [52-54]. Data on other effector cytokines of Th17 cells, such as *Il17a*, were not available in our differential gene list, probably resulting from the very low gene expression levels of Th17 effector cytokines. The observation that *Ccr6* shows decreased expression in the TNBS-treated ileum, together with the increased expression of *Il6*, could be indicative of increased Th17 effector responses and decreased Foxp3-induced regulatory responses in the TNBS-treated ileum [55,56]. Furthermore, some of the selected genes induced in Th17 cells compared to Th0 cells, such as the above-mentioned *Ccl9* and *Tnfrsf12A*, showed increased expression in this condition [57]. Taken together, the TNBS-treated ileum shows gene profiles indicative of Th17 cell priming. It has been reported earlier that CCL9 is involved in the recruitment of CD11b + dendritic cells (DCs) and that CD11b + DCs preferentially induce Th17 development [53,58,59]. Probably in keeping with this finding, is our observation that *Ccl9* together with integrin α M (*Itgam*/*Cd11b*) is increasingly expressed, which points to a role of CD11b + DCs in the TNBS-treated ileum of Th17 cell development. Furthermore, the increased expression of *S100A8* and *S100A9* genes in this model is most likely related to neutrophil abundance and chemotactic effects related to innate immunity, which in turn might be linked to Th17 cell responses, since neutrophils are considered to be the prime effector cells and inducers of Th17 differentiation [60,61]. We further observed a decreased expression of transcription factors T-box 21 (*Tbx21*/*Tbet*), as well as of the preferential receptor for Th1 cells, *Cxcr3*. In the TNBS-treated ileum, these changes suggest inhibition of Th1 cell development in favor of Th17 cell development, whereby Th17 cells further inhibit the Th1 cell development [52,62-64]. About the chemokines and their receptors, increased expression of *Cxcl4*, *Cxcr3*, *Ccl2-4*, *Ccl9* point to recruitment of immature DCs, T cells, monocytes or neutrophils [65], while other members of this group (*Cxcl9*, *Cxcl12*, *Ccl5*, *Ccl25* and *Ccr6*) exhibited a decreased expression. The repressed expression of *Cxcl9* is probably related to the downregulated Th1 responses, which again is in agreement with the observation that Th17 cells suppress Th1 cell differentiation [52]. The decreased expression of *Ccl25* is probably associated with the reduced homing of T cells and IgA + plasma cells to the intestine, which is consistent with suppression of the intestinal immune network for IgA production in this model, as will be discussed later [65]. Another interesting change that we observed in relation to the cytokines in this model, was the increased expression of *Il33*, a ligand for the IL1 family receptor T1/ST2. IL33 is mainly known as an inducer of Th2 differentiation, but has recently been attributed crucial functions in amplifying mucosal innate rather than acquired immune responses [66,67]. Moving on to regulatory mechanisms, increased expression of the Il1 receptor antagonist *Il1rn* in this model, in the light of elevated *Il1b* expression, supports the view of host mechanisms for the regulation of the IL1 system, as also observed in IBD [54]. The increased expression of *Thbs1* encoding thrombospondin 1 (THBS1) suggests that THBS1 is involved in the clearance of inflammatory cells in this model [68]. The increased expression of the regulatory cytokine *Il10*, retinol-binding protein 1 (*Rbp1*) and retinol dehydrogenase 10 (*Rdh10*) might be relevant in the light of a recent study demonstrating that during inflammatory conditions, Th17 cells of the regulatory phenotype (rTh17) also expressed high levels of IL10 and that the control of Th17 cells occurs in the small intestine [69]. These data suggest the in vivo presence of immunosuppressive activity and reciprocal regulatory responses in the TNBS-treated ileum, and hence point to the presence of host mechanisms that tune down exaggerated pro-inflammatory Th17 responses, in an attempt to reinforce intestinal homeostasis [52,70]. These findings are in line with previous studies, in which C57BL/6 and other mouse strains were used and which demonstrated that acute TNBS inflammation is self-limiting, with a naturally occurring healing response. This healing response was shown to involve the production of regulatory cytokines, which thereby cause the inflammation to eventually reach near complete remission shortly after the acute stage [21,27].

### Intestinal immune network for IgA production pathway and immune response category group

(Additional file 1, Additional file 2, Additional file 7: Tables S1 and Additional file 8: Table S2).

In the *S. mansoni*-infected ileum, our results demonstrate that the intestinal immune network for IgA production pathway and immune response category group is upregulated, conferring defense against toxins and microorganisms. The category group shared 3 genes (*Il4*, *Itgb7*, *Tnfsf13b*), all of which showed increased expression. Using in vitro methods, Crabtree et al. reported the increased immunoglobulin production in *S. mansoni* infections [71]. Probably related to these observations is the increased expression of the immunoglobulin heavy chain related gene *Ighv6-6* in the *S. mansoni*-infected ileum.

In the TNBS-treated ileum, our findings suggest that protective immune responses, the antibody line of defense and epithelial integrity are affected, leading to insufficient defense against microorganisms. The category group shared 10 genes (*Ccl25*, *Cxcl12*, *H2-Aa*, *H2-Ab1*, *H2-DMa*, *H2-DMb1*, *H2-DMb2*, *H2-Eb1*, *H2-Ob*, *Tnfsf13b*), all of which showed decreased expression. Several other important genes in this functional category group, such as the polymeric immunoglobulin receptor (*Pigr*), interferon regulatory factors, immunoglobulin-related genes immunoglobulin heavy chain complex (*Igh*), *Igh-2* and *Igh-6,* oligoadenylate synthetases, T-cell specific GTPases and immunity-related GTPase family, M (*Irgm*), were also found to display decreased expression. The polymeric immunoglobulin receptor and/or the secretory immunoglobulins have been reported to be important in the maintenance of epithelial integrity and mucosal homeostasis in dextran sulfate sodium (DSS)-induced mouse model of colitis [72].

### Complement and coagulation cascades pathway and protein activation cascade category group

**Figure 3:**
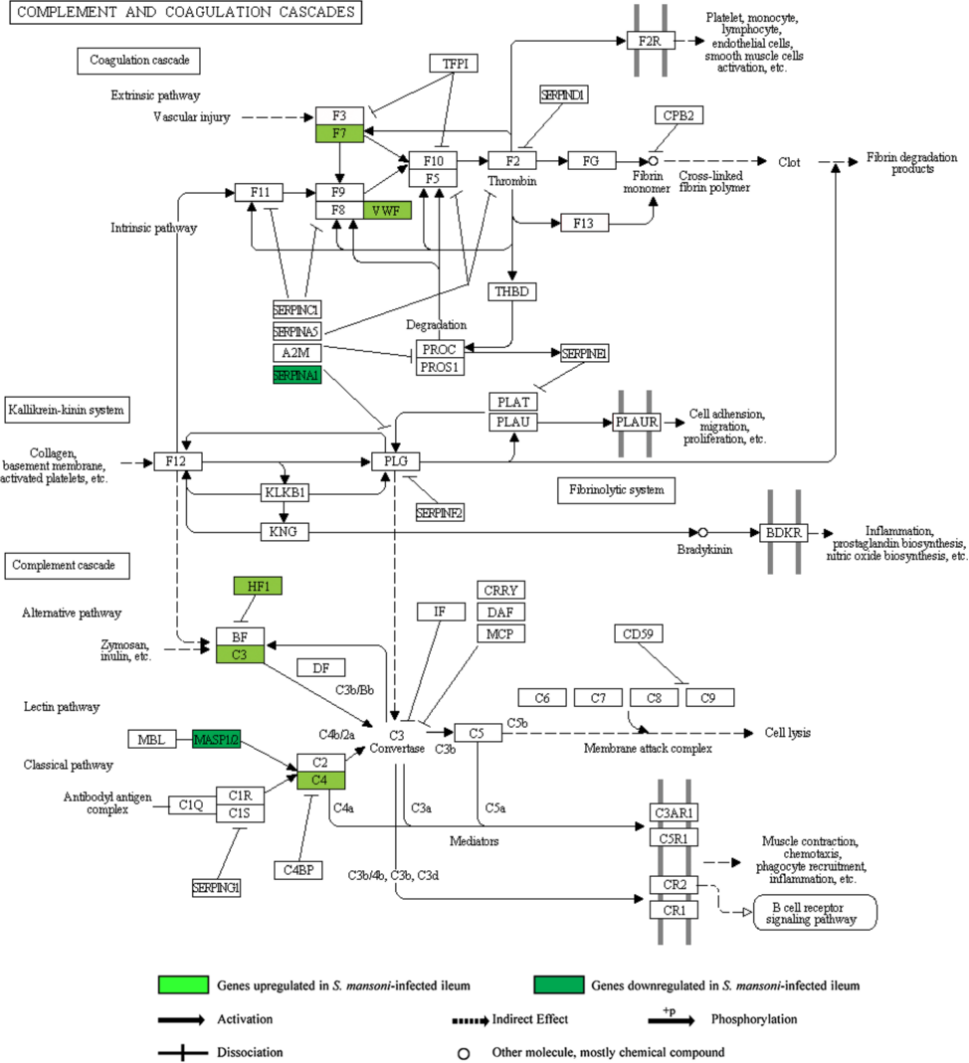
**Illustration of the KEGG complement and coagulation cascades pathway with location of 10 associated genes that were differentially expressed in the*S. mansoni *-infected ileum.**

(Additional file 1, Additional file 7: Table S1) (Figure 3).

In the *S. mansoni*-infected ileum, changes in this category group suggest complement activation, enhanced innate immune responses, coagulation activation, altered vascular homeostasis and affected acute phase responses, as also observed in some other animal models of intestinal inflammation [73,74]. The category group shared 4 genes (*C3*, *C4b*, *Cfh*, *F7*), all of which showed increased expression. The changes in complement component factor h (*Cfh*) and complement components 3 and 4 (*C3*, *C4b*), indicate enhanced innate immune responses [75,76]. C3 has also been proposed to have roles in directly triggering the degranulation of mast cells, increasing vascular permeability and in smooth muscle contraction [77]. Perhaps related to this complement cascade is our observation of the highly increased expression of long pentraxin (*Ptx3*), which is known to be produced by innate immune cells and involved in tuning complement activation and inflammation [78]. Furthermore, the increased expression of coagulation genes Von Willebrand factor homolog (*Vwf*) and coagulation factor VII (*F7*) implies enhanced coagulation, whereas the decreased expression of the serine peptidase inhibitor genes and regenerating islet-derived 3 alpha (*Reg3a*) suggests affected acute phase responses, since these genes fall under the acute phase response GO category.

In the TNBS-treated ileum, this category group did not show significant changes.

### ECM-receptor interaction pathway and cell adhesion category group

**Figure 4:**
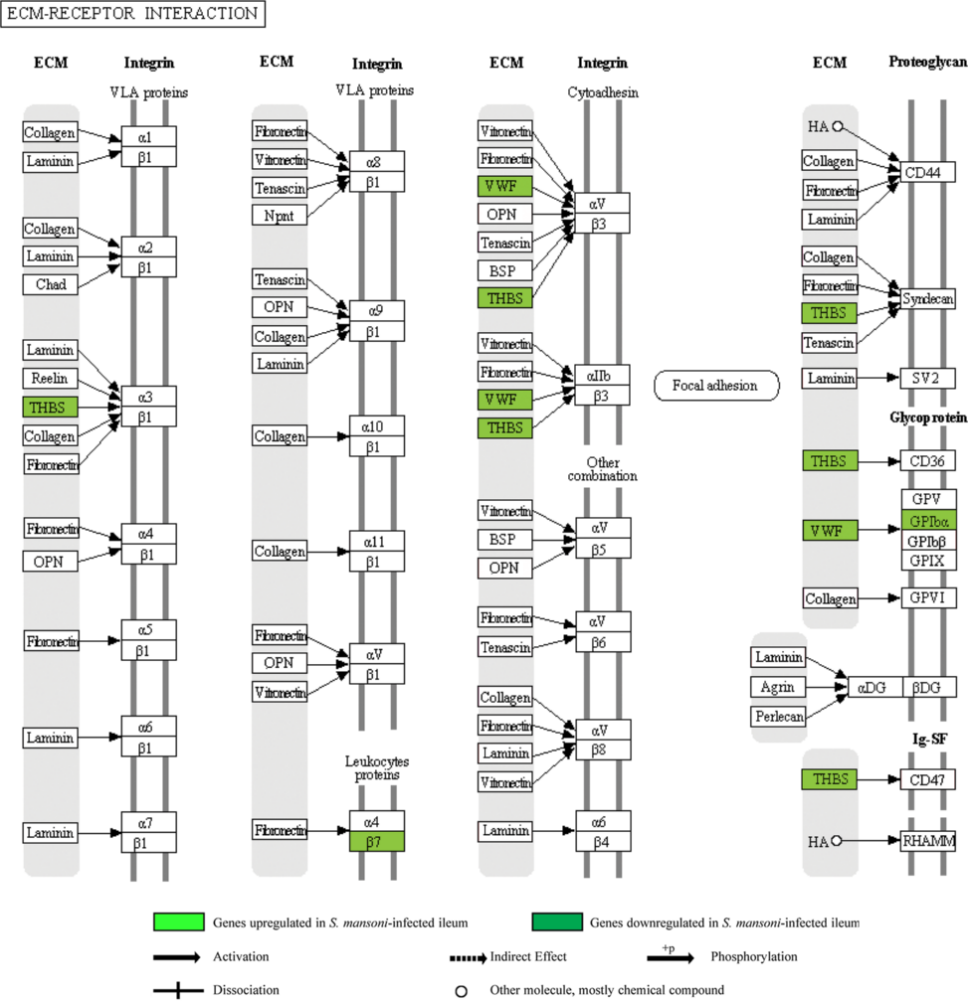
**Illustration of the KEGG extracellular matrix-receptor interaction pathway with location of 5 associated genes that were differentially expressed in the *S. mansoni*-infected ileum**

(Additional file 1, Additional file 7: Table S1) (Figure 4).

In the *S. mansoni*-infected ileum, our results pertaining to this category group suggest altered vascular homeostasis, recruitment of several types of immune cells, enhanced ECM turnover leading to altered adhesion, differentiation, apoptosis, and proliferation, enhanced tissue repair and defects in the epithelial barrier. The ECM-receptor interaction pathway-cell adhesion category group is upregulated in this condition and shared 5 genes (*Gp1ba*, *Itgb7*, *Thbs3*, *Thbs4*, *Vwf*), all of which showed increased expression. Increased expression of *Vwf* and the VWF-receptor gene glycoprotein 1b, alpha polypeptide (*Gp1ba*) is mainly suggestive of destabilization of the endothelial barrier, increased vascular permeability and promotion of the inflammatory response [68,74]. The increased expression of integrins *Itgae*/*Cd103* and *Itgb7* (*αEβ7*) suggests intraepithelial retention of immune cells in the *S. mansoni*-infected ileum [79]. As far as other genes in this category group are concerned, the increased expression of integrins *Itgae*//*Cd103* and *Itgax* (*Cd11c*) in this model indicates enhanced DC activity [80,81]. However, another study reported that CD103, despite displaying an upregulation, is dispensable for intestinal immunity during helminth infection [82]. Increased expression of *Siglec5* points to the accumulation and survival of eosinophils [83] and increased expression of collagen-related gene *Col18a1* points to preservation of integrity of ECM in the basement membrane and hence in wound healing processes in the *S. mansoni*-infected ileum [84]. Interestingly, two tight junction protein genes claudin 2 and 8 (*Cldn2*, *Cldn8*), which are related to cell adhesion, showed decreased expression in this model, indicating alterations in the epithelial paracellular transport [85], which could be associated with the previously reported impaired epithelial barrier function and altered permeability during *S. mansoni* infection [86].

In the TNBS-treated ileum, this category group did not show significant changes.

### FcεRI signaling pathway and regulation of acute inflammatory response category group

(Additional file 1, Additional file 7: Table S1).

In the *S. mansoni*-infected ileum, FcεRI signaling pathway-regulation of acute inflammatory response category group is upregulated, suggesting enhanced stimulation and amplification of mast cell responses and acute inflammatory responses, such as the Th2 responses, conferring resistance to schistosome eggs. This particular category group shared genes, such as *Fcer1a* and *Il4,* and these genes showed increased expression. Fc receptor IgE high affinity I α polypeptide (*Fcer1a*), is known to encode the high-affinity IgE receptor subunit α, and to be expressed on mast cells and granulocytes [87]. Acute schistosomiasis is characterized by strong host immune responses with elevated IgE levels correlating with the magnitude of the infection [88]. The interaction of antigen with IgE bound to FcεRI triggers mast cells to degranulate, resulting in the release of increased levels of mediators that modulate immune responses and increase smooth muscle contraction and vascular permeability [89]. In line with these findings, we observed increased expression of several mast cell-associated genes, such as the mast cell chymases 1, 2 (*Cma1*, *Cma2*), and the mast cell proteases *Mcpt1*, *Mcpt2*, *Mcpt4* and *Mcpt9* in this model, along with the afore-mentioned *Fcer1a*. These findings corroborate the results of another study that reported the predominant recruitment of mucosal mast cells expressing mouse mast cell protease 1 (mMCP-1), a protein encoded by *Mcpt9*, in acute intestinal schistosomiasis [90]. A crucial role for chymase immunopositive-mast cells has also been reported in the pathophysiology of CD [91]. A positive feedback mechanism between FcεRI signaling and IL4 production has been described, and indeed, as discussed earlier, *Il4* was increasingly expressed in the *S. mansoni*-infected ileum [92].

The TNBS-treated ileum did not reveal any significant changes related to this category group, which is not surprising given the known role of the Fcε receptor in allergic reactions and defense against parasitic infections, which is not the case in acute TNBS-induced ileitis.

### Antigen processing and presentation pathway and antigen processing and presentation (of endogenous antigen, of exogenous peptide antigen and of peptide or polysaccharide antigen via MHC class II) category group

**Figure 5:**
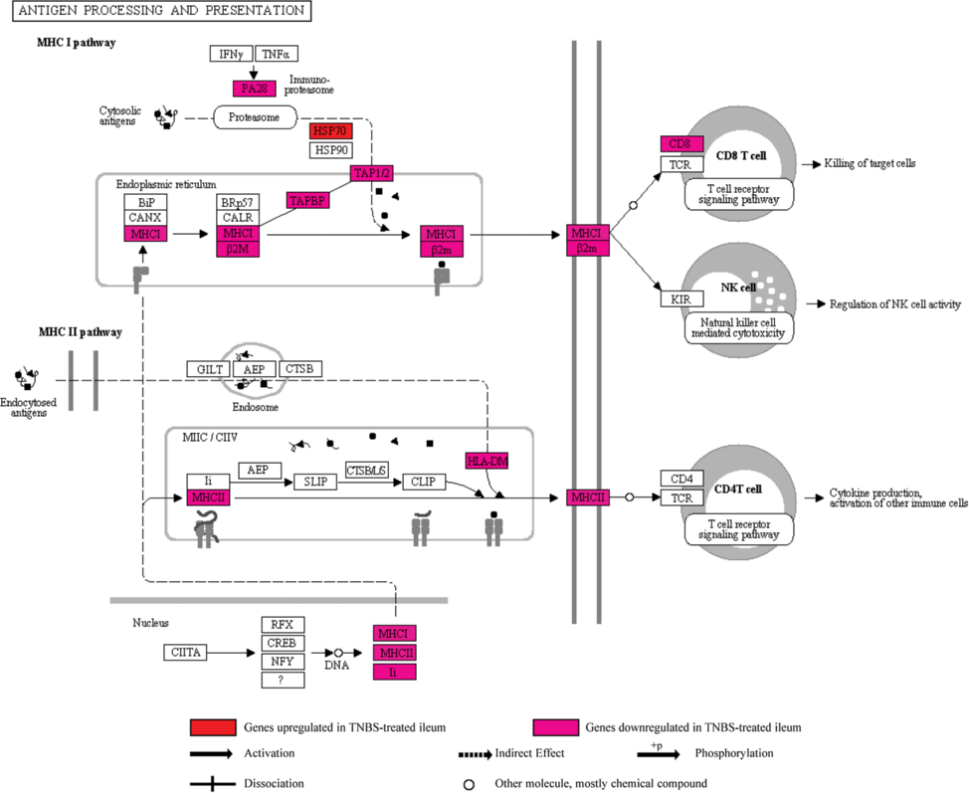
Illustration of the KEGG antigen processing and presentation pathway with location of 20 associated genes that were differentially expressed in the TNBS-treated ileum.

(Additional file 2, Additional file 8: Table S2) (Figure 5).

In the *S. mansoni*-infected ileum, no significant changes were observed in this category group.

In the TNBS-treated ileum, the altered gene expression observed in this category group indicates that antigen processing and presentation are defective in acute TNBS-induced ileitis. This category group shared 13 genes (*H2-Aa*, *H2-Ab1*, *H2-D1*, *H2-DMa*, *H2-DMb1*, *H2-DMb2*, *H2-Eb1*, *H2-K1*, *H2-Ob*, *H2-Q7*, *H2-T23*, *Tap2*, *Tapbp*), all of which showed decreased expression. Apart from the above-mentioned MHC class I and class II antigen genes, other genes in this category group, such as *Cd8a* T cell gene and the T cell receptor genes *Tcra* and *Tcrg-V4* also showed decreased expression. The earlier reported downregulation of histocompatibility 2, class II antigen E β (*H2-Eb1*) and of T cell receptor γ in a C57BL/6 mouse model of TNBS-induced colitis is therefore in agreement with our observations [93]. These changes point to defective antigen presentation, decreased cytotoxic T cell function and the suppression of unconventional regulatory T cells, such as the resident TCRγδ or TCRαβ CD8+ intraepithelial lymphocytes [94]. The decreased expression of heat shock protein 2 (*Hspa2*), which encodes HSP70A2*,* along with the increased expression of *Hspb1* (HSP25), is indicative of a change in the inherent ability of epithelial cells to defend themselves against cellular stress and injury in acute TNBS-induced ileitis. *Hspa2* downregulation also hints at decreased antigen presentation to CD4+ T cells by antigen presenting cells [95].

### Cell adhesion molecules pathway and cell adhesion category group

(Additional file 2, Additional file 8: Table S2).

In the *S. mansoni*-infected ileum, no significant changes were observed in this category group.

In the TNBS-treated ileum, changes in this category group suggest affected integrity and function of the intestinal epithelial barrier and altered recruitment of several types of immune cells. This particular category group shared 8 genes (*Cd6*, *Cldn2*, *Cldn8*, *Itga8*, *Itgb7*, *Negr1*, *Neo1*, *Ptprf*), all of which showed decreased expression. Changes related to these claudin genes have also been described in human IBD and in ileitis models [85,96]. Decreased expression of integrin beta 7 (*Itgb7*) together with the afore-mentioned *Ccl5* in this model indicates suppression of lymphocyte recruitment to the intestine [97]. Furthermore, changes in some other genes in this category group, such as the decreased expression of *Itgae*/*Cd103*, together with increased expression of *Itgam*, and of the afore-mentioned *Ccl9*, indicate the loss of CD103+ intestinal DCs, as seen in murine colitis models [98], and an increased efflux of activated macrophages and DCs to the lymphatics. These changes are perhaps instrumental in the restoration of homeostatic balance through T cell induction in the TNBS-treated ileum [59,99]. Increased expression of genes encoding proteins such as laminin and decreased expression of genes encoding collagens, integrins α11, and tenascin in the cell adhesion category are mainly suggestive of compositional changes in the epithelial basement membrane and interstitial matrix and suppressed wound healing [100,101].

### TLR signaling pathway and immune, inflammatory response category group

(Additional file 2, Additional file 8: Table S2).

In the *S. mansoni*-infected ileum, this TLR-associated category group did not show differential expression, which is in accordance with previous reports stating that antigen secretions of schistosoma eggs, such as the soluble egg antigen, do not require the expression of TLR2 or TLR4 on DCs to induce a polarized Th2 response [102]. Instead, pattern recognition receptor genes other than TLRs, such as the C-type lectin family member genes showed increased expression, confirming that C-type lectins are likely involved in innate signaling in acute intestinal schistosomiasis [102].

In the TNBS-treated ileum, changes in this category group suggest altered innate immune responses, suppressed barrier protection and aggravated inflammatory responses. The category group shared 6 genes that showed increased expression (*Ccl3*, *Ccl4*, *Cd14*, *Il1b*, *Il6*, *Tlr6*) and 5 shared genes that showed decreased expression (*Ccl5*, *Cxcl9*, *Irf7*, *Tlr1*, *Tlr3*). Upregulation of TLR6 has been suggested to lead to pathogen recognition and rapid activation of innate immunity by the induction of inflammatory molecules [103]. The increased expression of *Il6*, *Il1b, Ccl3* (*Mip-1α*), *Ccl4* (*Mip-1β*) is probably related to this finding. TLR1 expression has been shown to be downregulated in human inflamed colonic epithelial and lamina propria cells and has been linked to responses to TLR2 ligands [104]. A protective role for TLR3 signaling has been put forward in the pathogenesis of intestinal inflammation in a murine acute colitis model [105]. Therefore, the observed decreased expression of *Tlr3* in our study probably indicates suppression of protective TLR3 signaling. Another interesting gene in this functional category group, which was found to be highly expressed in the TNBS-treated ileum, is secreted phosphoprotein 1 (*Spp1*), and the protein encoded by this gene is known to be a key immune modulator, stimulating stress responses as well as both pro- and anti-inflammatory processes [106].

### ABC transporters pathway and response to chemical stimulus category group

**Figure 6:**
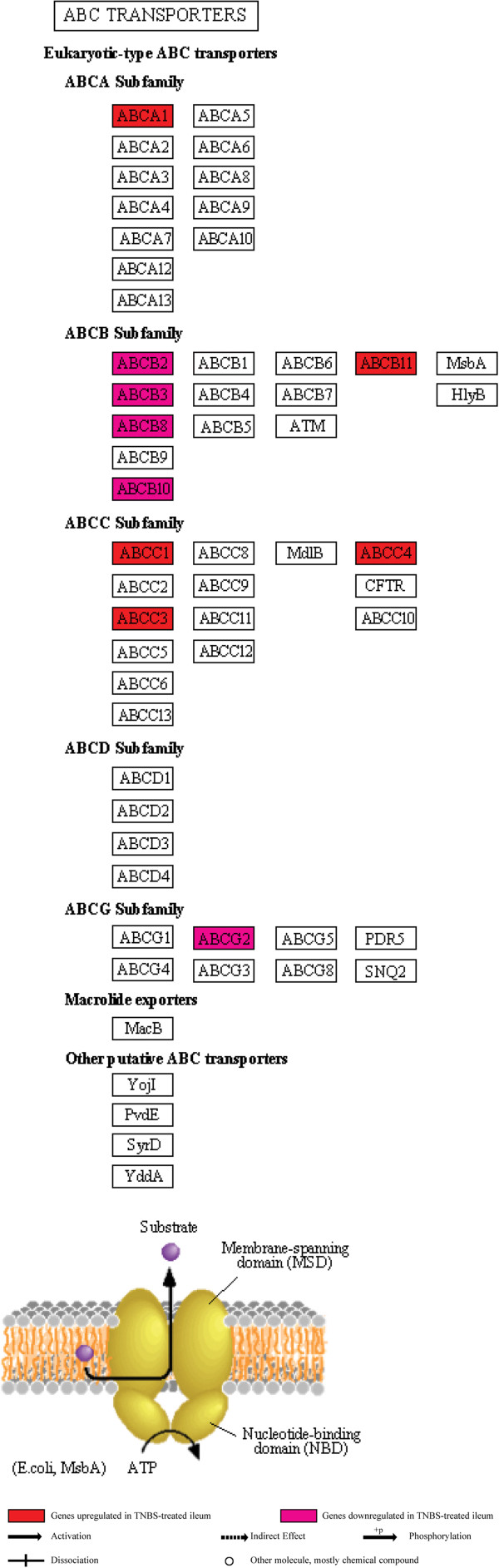
Illustration of the KEGG ABC transporters pathway with location of 14 associated genes that were differentially expressed in the TNBS-treated ileum.

(Additional file 2, Additional file 8: Table S2) (Figure 6).

In the *S. mansoni*-infected ileum, this category group did not show significant changes.

In the TNBS-treated ileum, changes in this category group suggest a defective efflux of pathogens, drugs and toxins, thereby an impaired epithelial barrier function, an enhanced response to chemical stimuli and altered vascular homeostasis. This category group shared 3 genes (*Abca1*, *Abcb11*, *Abcc1*) with increased expression. Other genes in this functional category group that showed decreased expression included *Abca1*, *Abcb10*, *Abcb11*, *Abcc1*, *Abcc3*, *Abcc4*, *Abcg2*, *Tap1* and *Tap2*. The ATP-binding cassette sub-family B member 1, (*Abcb1a*), which has been observed to change expression in models of colitis and CD [4,107], was not found to be differentially expressed. Other changes related to this category group included those of the coagulation cascade, which seems to be activated given the increased expression of coagulation factors III and XIII (*F3*, *F13*), the plasminogen activator, the urokinase receptor (*Plaur*) and some serine (or cysteine) peptidase inhibitor members. These changes lend further link to previous studies that have demonstrated that TNBS administration causes bleeding within the ileal wall [30] and to the existing role of coagulation in the perpetuation of inflammation, as also reported in human and experimental IBD [68,73].

### Comparison of the differentially expressed genes in our study with IBD

Comparison of the differentially expressed genes in the *S. mansoni*-infected ileum with 32 genes that have previously been shown to exhibit an altered expression pattern in murine models of IBD [4] yielded only 1 overlapping gene (1 upregulated) in the *S. mansoni*-infected ileum; i.e., *Timp1*. This change in *Timp1* expression might be related to an altered epithelial barrier and tissue remodeling [108]. It can be inferred from the comparison that the *S. mansoni*-infected ileum, which is a model of intestinal inflammation not related to IBD, hardly shows any similarity with IBD-associated gene expression. The change in *Timp1* expression was also observed in the TNBS-treated ileum (see below), implying that activity of this gene is probably not specific to the inflammation type.

In the TNBS-treated ileum, however, comparison with the above-mentioned 32 genes [4], yielded 14 overlapping genes (12 upregulated and 2 downregulated). The higher expressed genes were *Il6*, *Ccl2*, *Ccl3*, *Ccl4*, *Cxcl5*, *Mmp3*, *Mmp7*, *Mmp9*, *Timp1*, *S100a8*, *S100a9* and *Ptgs2* and the lower expressed genes were *Ccl5* and *Cxcr3*. Increased expression of *Il6*, chemokines and neutrophil-associated genes, are indicative of pro-inflammatory responses and the recruitment of macrophages and granulocytes, as mentioned above. Changes in the expression of matrix metalloproteinases and of the tissue inhibitor of metalloproteinase 1 (*Timp1*) are probably related to tissue injury, tissue remodeling, angiogenesis, and promotion of leukocyte extravasation observed in this model [108]. Increased expression of *Ptgs2* points to prostaglandin synthesis, as suggested in colonic epithelial cells in IBD and in experimental colitis [4]. It can be inferred from the comparison that although our TNBS-induced ileitis model is an acute insult, 14 genes (out of the 32 genes previously known to show an altered expression pattern in murine models of IBD [4]) showed changed expression; and as such, the expression profile in this model bears considerable relevance to that of IBD. A study of murine TNBS-induced inflammation in colon, conducted by another group, however, revealed very few differentially expressed genes and did not show significant similarities with the expression profile in human IBD [4]. The divergent results in this respect between the latter and our study may originate from several factors, such as the use of a different mouse strain, the application of different dosages, the region studied (colon vs. ileum,) and the type of inflammation (only delayed type hypersensitivity reaction after administration of a second dose vs. acute inflammation in our study). Our study, compared to previously published studies such as the one mentioned above [4], as well as some others [109], highlight the significant differences of results in gene expression in the same inflammatory condition, i.e., in TNBS-induced inflammation, but in other regions of the GI tract and therefore strengthen the need of studies such as ours, to account for the region-specific differences. Furthermore, comparison of the differentially expressed genes in our study with the list of 77 candidate genes located within IBD susceptibility loci [109-112] did not yield any overlapping genes in the *S. mansoni*-infected ileum, whereas 9 genes (4 up and 5 down) were overlapping in the TNBS-treated ileum (Table 1).

**Table 1:** **Comparison of the differentially expressed genes in the*S. mansoni*-infected and the TNBS-treated ileum with candidate genes located within IBD susceptibility loci**

**Gene symbol**	**IBD type**	***S. mansoni*-infected ileum**	**TNBS-treated ileum**
*NOD2*	CD	ND	ND
*ATG16*	CD	ND	ND
*IRGM*	CD	ND	↓
*LRRK2*	CD	ND	ND
*PTPN22*	CD	ND	ND
*CCR6*	CD	ND	↓
*IL-2RA*	CD	ND	ND
*IL-18RAP*	CD	ND	ND
*IL-27*	CD	ND	ND
*ERAP2* (*Erap1*/*Eraap*)	CD	ND	ND
*ITLN1*	CD	ND	ND
*CCL2* (*Ccl12*)	CD	ND	ND
*CCL7*	CD	ND	ND
*TNFSF11*	CD	ND	ND
*BACH2*	CD	ND	ND
*TAGAP*	CD	ND	ND
*VAMP3*	CD	ND	ND
*DENNDIB*	CD	ND	ND
*DNMT3A*	CD	ND	ND
*GCKR*	CD	ND	ND
*THADA*	CD	ND	ND
*SP140*	CD	ND	ND
*PRDX5*	CD	ND	ND
*ZFP36L1*	CD	ND	ND
*ZMIZ1*	CD	ND	ND
*MUC1*	CD	ND	ND
*SCAMP3*	CD	ND	ND
*CPEB4*	CD	ND	ND
*FADS1*	CD	ND	ND
*SLC22A5*	CD	ND	ND
*ECM1*	UC	ND	ND
*HNF4A*	UC	ND	ND
*CDH1*	UC	ND	ND
*LAMB1*	UC	ND	ND
*GNA12*	UC	ND	ND
*IFNG*	UC	ND	ND
*IL-26 (Il-22)*	UC	ND	ND
*IL-8RA/IL-8RB*	UC	ND	↑
*IL-2*	UC	ND	ND
*IL-21*	UC	ND	ND
*IL-7R*	UC	ND	ND
*TNFRSF9*	UC	ND	ND
*TNFRSF14*	UC	ND	↓
*FCGR2A* (*Fcgr3*/*Fcgr2b*)	UC	ND	ND
*IRF5*	UC	ND	ND
*LSP1*	UC	ND	ND
*OTUD3*	UC	ND	ND
*PLA2G2E*	UC	ND	ND
*DAP*	UC	ND	ND
*PIM3*	UC	ND	ND
*CAPN10*	UC	ND	ND
*IL-23R*	CD/UC	ND	ND
*IL-12B*	CD/UC	ND	ND
*STAT3*	CD/UC	ND	↑
*AK2*	CD/UC	ND	ND
*TYK2*	CD/UC	ND	ND
*NKX2-3*	CD/UC	ND	ND
*CREM*	CD/UC	ND	ND
*C11Orf30* (*2210018M11Rik*)	CD/UC	ND	ND
*ORMDL3*	CD/UC	ND	ND
*RTEL1*	CD/UC	ND	ND
*PTGER4*	CD/UC	ND	↓
*KIF21B*	CD/UC	ND	ND
*CDKAL1*	CD/UC	ND	ND
*ZNF365* (*Zfp365*)	CD/UC	ND	ND
*HLA-DRB1* (*H2-Eb1*)	CD/UC	ND	↓
*MST1*	CD/UC	ND	ND
*IL-10*	CD/UC	ND	↑
*CARD9*	CD/UC	ND	ND
*REL*	CD/UC	ND	ND
*PRDM1*	CD/UC	ND	ND
*TNFSF15*	CD/UC	ND	ND
*ICOSLG* (*Icosl*)	CD/UC	ND	ND
*IL-1R2*	CD/UC	ND	↑
*YDJC* (*1810015A11Rik, Ccdc116*)	CD/UC	ND	ND
*SMAD3*	CD/UC	ND	ND
*PTPN2*	CD/UC	ND	ND

The table compares candidate genes within IBD susceptibility loci to the differentially expressed genes in the *S. mansoni*-infected and in the TNBS-treated ileum. Gene symbol denotes the gene symbol in human along with the murine homologue where applicable in brackets. CD, human Crohn's disease; UC, human ulcerative colitis; ND, no difference; ↑, upregulated; ↓, downregulated. No genes were overlapping in the *S. mansoni*-infected ileum, while 9 genes were overlapping in the TNBS-treated ileum.

This brief comparative analysis with IBD-related or IBD-associated genes, clearly demonstrates that gene expression aspects of the TNBS-induced ileitis model in our study have noticeable relevance to IBD.

## Conclusions

The present study in mouse is the first to identify changes in whole-genome gene expression in the acutely inflamed ileum of two murine models of intestinal inflammation and to characterize the biological relevance related to these changes. The results demonstrate that gene expression profiles and patterns are considerably distinct in the two models owing to the differences in etiology. The results suggest that acute intestinal schistosomiasis shows enhanced Th2 responses, whereas acute TNBS-induced ileitis exhibits Th17 cell priming. Although two functional category groups (cytokine-cytokine receptor interaction pathway-immune response category group and intestinal immune network for IgA production pathway-immune response category group) are common to both models of inflammation, the predisposing genes are largely unique within the pathways. Other functional category groups, such as the complement and coagulation cascades pathway-protein activation cascade, ECM-receptor interaction pathway-cell adhesion, Fc epsilon RI signaling pathway-acute inflammatory response are unique to the acute intestinal schistosomiasis model, and are upregulated, whereas antigen processing and presentation, cell adhesion, TLR signaling pathway-immune and inflammatory response and ABC transporters pathway-response to chemical stimulus category groups are unique to the acute TNBS-induced ileitis model, and are more bent towards downregulation. All the above findings underscore the distinct gene expression patterns in the two inflammation models of our study. Acute intestinal schistosomiasis seems to promote a number of specific processes, such as complement-mediated innate immunity, protein activation, ECM turnover, acute inflammatory responses as well as antibody-mediated defense responses. Acute TNBS-induced ileitis seems to be associated with aspecific proinflammatory responses, alterations in TLR-mediated innate immunity, defective antigen processing and presentation, angiogenesis and anti-angiogenesis, reduced cytotoxic T cell function, compositional changes in the epithelial basement membrane and interstitial matrix, enhanced tissue remodeling and a defective antibody line of defense. Altered vascular homeostasis and impaired epithelial barrier function seem to be common features in both models of inflammation. The gene expression profiles in acute TNBS-induced ileitis show considerable relevance to those of IBD. This is of course no complete resemblance, but insight into the differences between animal models and the human situation is equally important. Furthermore, better characterization of animal models leads to better interpretation of results concerning translational research. In addition to the above findings, the present study revealed a differentially expressed gene list that includes many genes for which pathways or functional annotations have not yet been defined. Therefore, the comprehensive differential gene list together with the functional grouping, in particular, provided by this study offers a valuable resource and a starting point to explore extended functional networks, new targets or a specific novel pathway in greater detail related to small bowel inflammation. Future application of these analyses to other ileal inflammation models, including chronic stages and other time points, will yield greater insights and lead to a better understanding of ileal inflammation.

## Abbreviations

CD: human Crohn's disease; DCs: dendritic cells; ECM: extracellular matrix; FcεRI: Fc epsilon receptor I; GI: gastrointestinal; GO: gene ontology; IBD: inflammatory bowel diseases; IgA: immunoglobulin A; KEGG: Kyoto Encyclopedia for Genes and Genomes; log_2_FC: log_2_ fold change; MHC: major histocompatibility complex; TLR: Toll-like receptor; TNBS: 2,4,6-trinitrobenzene sulfonate; UC: human ulcerative colitis.

## Competing interests

The authors declare that they have no competing interests.

## Authors’ contributions

LRA, DK, RB and LV conceived the study, designed the experiments, prepared the samples and performed the experiments. LRA, DK and RB conducted the data analysis and wrote the initial manuscript. LRA, LV, DA, LVN and JPT contributed to the bioinformatics analysis and manuscript editing. LVN and JPT provided critical advice and conceptual guidance for the study. All authors read and approved the final manuscript.

## Supplementary Material

Additional file 1 **Complete list of differentially expressed genes in the *S. mansoni*-infected ileum.** Differentially expressed genes in the *S. mansoni*-infected ileum are represented with their ID (Agilent probe ID), GB_ACC (gene bank accession), GENE_SYMBOL (gene symbol), GENE_NAME (gene name), log_2_FC (log_2_ fold change), p-value and adjusted p-value. Upregulated genes are shown in red; downregulated genes are shown in green; overlapping genes that changed in the same direction as in the TNBS-treated ileum are displayed in bold; overlapping genes that changed in the opposite direction to that of the TNBS-treated ileum are underlined. All genes listed in this file showed significant differential expression (p-value < 0.05, adjusted p-value < 0.1 and log_2_ fold change > 1 or < −1) relative to controls. Click here for file

Additional file 2 **Complete list of differentially expressed genes in the TNBS-treated ileum.** Differentially expressed genes in the TNBS-treated ileum are represented with their ID (Agilent probe ID), GB_ACC (gene bank accession), GENE_SYMBOL (gene symbol), GENE_NAME (gene name), log_2_FC (log_2_ fold change), p-value and adjusted p-value. Upregulated genes are shown in red; downregulated genes are shown in green; overlapping genes that changed in the same direction as in the *S. mansoni*-infected ileum are displayed in bold; overlapping genes that changed in the opposite direction to that of the *S. mansoni*-infected ileum are underlined. All genes listed in this file showed significant differential expression (p-value < 0.05, adjusted p-value < 0.1 and log_2_ fold change > 1 or < −1) relative to controls. Click here for file

Additional file 3 **Complete list of differentially expressed genes that could be annotated with a KEGG pathway term and/or a GO term in the *S. mansoni*-infected ileum.** Differentially expressed genes are listed together with their KEGG and/or GO annotations. GenID (Ensembl ID), Symbol (gene symbol), #GO-Terms Associated (number of GO terms associated), Percent GO-Terms Associated, UID-str (unique Agilent ID), GO Names (names of associated terms). Of the 207 unique genes that were differentially expressed in the *S. mansoni*-infected ileum, 42 genes could be linked to a KEGG pathway term and 144 genes could be linked to a GO term. Click here for file

Additional file 4 **Complete list of differentially expressed genes that could be annotated with a KEGG pathway term and/or a GO term in the TNBS-treated ileum.** Differentially expressed genes are listed together with their KEGG and/or GO annotations. GenID (Ensembl ID), Symbol (gene symbol), #GO-Terms Associated (number of GO terms associated), Percent GO-Terms Associated, UID-str (unique Agilent ID), GO Names (names of associated terms). Of the 1417 unique genes that were differentially expressed in the TNBS-treated ileum, 232 genes could be linked to a KEGG pathway term and 1050 genes could be linked to a GO term. Click here for file

Additional file 5 **KEGG pathways and GO (biological process) categories that were over-represented in the *S. mansoni*-infected ileum.** KEGG pathways and GO (biological process) categories that were over-represented in the *S. mansoni*-infected ileum are represented with their MAPP Name (KEGG pathway name), Z score (z-score), AdjustedP (adjusted permutation p-value calculated using the Benjamini-Hochberg method)), gene symbols, AVG-logFC (average log_2_FC for each enriched term calculated based on associated differential genes) and AVG-adj.P. Val (average adjusted p-value for each enriched term calculated based on associated differential genes, GOID (Gene ontology ID), GO Name (name of GO category). Threshold criteria for over-representation: z-score > 2, at least three genes from the input list in the enriched category, an adjusted permutation p-value ≤ 0.35. Top-scoring functional category groups for KEGG pathway and GO categories are shown in colour as follows: Cytokine-cytokine receptor interaction pathway and immune, defense response category group. Intestinal immune network for IgA production pathway and immune response category group. Complement and coagulation cascades pathway and protein activation cascade category group. ECM-receptor interaction pathway and cell adhesion category group. Fc epsilon RI signaling pathway and acute inflammatory response category. (Note: Although immune response category is common to more than one category group, it is coloured only once). Click here for file

Additional file 6 **KEGG pathways and GO (biological process) categories that were over-represented in the TNBS-treated ileum.** KEGG pathways and GO (biological process) categories that were over-represented in the TNBS-treated ileum are represented with their MAPP Name (KEGG pathway name), Z score (z-score), AdjustedP (adjusted permutation p-value calculated using the Benjamini-Hochberg method)), gene symbols, AVG-logFC (average log_2_FC for each enriched term calculated based on associated differential genes) and AVG-adj.P. Val (average adjusted p-value for each enriched term calculated based on associated differential genes, GOID (Gene ontology ID), GO Name (name of GO category). Threshold criteria for over-representation: z-score > 2, at least three genes from the input list in the enriched category, an adjusted permutation p-value ≤ 0.35. Top-scoring functional category groups for KEGG pathway and GO categories are shown in colour as follows: Cytokine-cytokine receptor interaction pathway and inflammatory, immune response category group. Intestinal immune network for IgA production pathway and immune response category group. Antigen processing and presentation pathway and antigen processing and presentation category group. Cell adhesion molecules pathway and cell adhesion category group. Toll-like receptor signaling pathway and immune and inflammatory response category group. ABC transporters pathway and response to chemical stimulus category group. (Note: Although immune, inflammatory response categories are common to more than one category group, they are coloured only once). Click here for file

Additional file 7 **Table S1.** High scoring functional category groups in the *S. mansoni*-infected ileum. Click here for file

Additional file 8 **Table S2.** High scoring functional category groups in the TNBS-treated ileum. Click here for file
