# Supplementary material for: Whole-genome microarray analysis and functional characterization reveal distinct gene expression profiles and patterns in two mouse models of ileal inflammation
Source: BMC Genomics. 2012 Aug 6;13:377. doi: 10.1186/1471-2164-13-377 (PMC3599598; doi:10.1186/1471-2164-13-377)
Supplement: Additional file 7 — Table S1. High scoring functional category groups in the S. mansoni-infected ileum. [file 1471-2164-13-377-S7.doc]

**Table 1** High scoring functional category groups in the *S. mansoni*-infected ileum.

| **KEGG pathways overrepresented in upregulated genes** | | | | | | |
| --- | --- | --- | --- | --- | --- | --- |
|  | **MAPP Name** | **Z Score** | **AdjustedP** | **gene symbols** | **AVG-logFC** | **AVG-adj.P.Val** |
|  | Complement and coagulation cascades | 5.80 | 0.04 | C3\|C4b\|Cfh\|F7\|Vwf | 1.72 | 0.05 |
|  | ECM-receptor interaction | 4.96 | 0.04 | Gp1ba\|Itgb7\|Thbs3\|Thbs4\|Vwf | 1.97 | 0.05 |
|  | Intestinal immune network for IgA production | 4.08 | 0.11 | Il4\|Itgb7\|Tnfsf13b | 1.75 | 0.06 |
|  | Fc epsilon RI signaling pathway | 2.81 | 0.33 | Fcer1a\|Il4\|Pik3r3 | 2.65 | 0.03 |
|  | Cytokine-cytokine receptor interaction | 2.77 | 0.05 | Ccl24\|Ccr5\|Il18r1\|Il4\|Il6ra\|Tnfsf13b | 2.37 | 0.06 |
| **KEGG pathways overrepresented in downregulated genes** | | | | | | |
|  | **MAPP Name** | **Z Score** | **AdjustedP** | **gene symbols** | **AVG-logFC** | **AVG-adj.P.Val** |
|  | Complement and coagulation cascades | 7.27 | 0.23 | Serpina1a\|Serpina1b\|Serpina1c\|Serpina1d\|Serpina1e | -1.73 | 0.085 |
| **GO categories overrepresented in upregulated genes** | | | | | | |
| **GOID** | **GO Name** | **Z Score** | **AdjustedP** | **gene symbols** | **AVG-logFC** | **AVG-adj.P.Val** |
| GO:0006955 | immune response | 8.30 | 0.09 | C3|C4b|Ccl24|Ccr5|Cfh|Clec4a2|Dhx58|Il18r1|Il1rl1|Il4|Mx2|Oas1a|Oas1e|Oas1f|Oas3|Prg2|Tgtp1|Tgtp2|Tnfsf13b | 1.84 | 0.06 |
| GO:0002673 | regulation of acute inflammatory response | 7.91 | 0.09 | C3|Ccr5|Cfh|Fcer1a|Il4 | 2.39 | 0.04 |
| GO:0006952 | defense response | 7.45 | 0.09 | C3|C4b|Ccl24|Ccr5|Cd163|Cela1|Cfh|Chi3l3|Chi3l4|Clec4a2|Dhx58|Hck|Il18r1|Il1rl1|Il6ra|Itgax|Mx2|Penk|Prg2 | 2.36 | 0.06 |
| GO:0072376 | protein activation cascade | 6.82 | 0.09 | C3|C4b|Cfh|F7 | 1.85 | 0.06 |
| GO:0007155 | cell adhesion | 4.82 | 0.09 | Amigo2|Col18a1|Cpxm1|Ctgf|Cyr61|Gp1ba|Itgae|Itgax|Itgb7|Muc4|Nlgn3|Siglec5|Thbs3|Thbs4|Vwf | 1.69 | 0.06 |

High scoring functional category groups (KEGG pathways + GO (biological process) categories) in the *S. mansoni*-infected ileum are shown in colour as follows: Cytokine-cytokine receptor interaction pathway and immune, defense response category group. Intestinal immune network for IgA production pathway and immune response category group. Complement and coagulation cascades pathway and protein activation cascade category group. ECM-receptor interaction pathway and cell adhesion category group. Fc epsilon RI signaling pathway and acute inflammatory response category. (Note: Although immune response category is common to more than one category group, it is coloured only once). MAPP Name (KEGG pathway name), Z score (z-score), AdjustedP (adjusted permutation p-value calculated using the Benjamini-Hochberg method)), gene symbols, AVG-logFC (average log_2_FC for each enriched term calculated based on associated differential genes) and AVG-adj.P.Val (average adjusted p-value for each enriched term calculated based on associated differential genes, GOID (Gene ontology ID), GO Name (name of GO category). Threshold criteria for over-representation: z-score>2, at least three genes from the input list in the enriched term, an adjusted permutation p-value≤0.35.
