# Supplementary material for: Whole-genome microarray analysis and functional characterization reveal distinct gene expression profiles and patterns in two mouse models of ileal inflammation
Source: BMC Genomics. 2012 Aug 6;13:377. doi: 10.1186/1471-2164-13-377 (PMC3599598; doi:10.1186/1471-2164-13-377)
Supplement: Additional file 8 — Table S2. High scoring functional category groups in the TNBS-treated ileum. [file 1471-2164-13-377-S8.doc]

**Table 2** High scoring functional category groups in the TNBS-treated ileum.

| **KEGG pathways overrepresented in upregulated genes** | | | | | | |
| --- | --- | --- | --- | --- | --- | --- |
|  | **MAPP Name** | **Z Score** | **AdjustedP** | **gene symbols** | **AVG-logFC** | **AVG-adj.P.Val** |
|  | Cytokine-cytokine receptor interaction | 3.50 | 0.009 | Ccl3\|Ccl4\|Ccl9\|Cxcl5\|Cxcr2\|Ifnar2\|Il10rb\|Il1b\|Il1r2\|Il24\|Il5ra\|Il6\|Kitl\|Lifr\|Osmr\|Pf4\|Ppbp\|Tnfrsf12a | 3.06 | 0.038 |
|  | Toll-like receptor signaling pathway | 3.26 | 0.009 | Ccl3\|Ccl4\|Cd14\|Ifnar2\|Il1b\|Il6\|Map2k7\|Spp1\|Tlr6 | 3.60 | 0.049 |
|  | ABC transporters | 3.05 | 0.042 | Abca1\|Abcb11\|Abcc1\|Abcc3\|Abcc4 | 1.58 | 0.037 |
| **KEGG pathways overrepresented in downregulated genes** | | | | | | |
|  | **MAPP Name** | **Z Score** | **AdjustedP** | **gene symbols** | **AVG-logFC** | **AVG-adj.P.Val** |
|  | Antigen processing and presentation | 7.94 | 0.01 | CR974466.3|Cd8a|H2-Aa|H2-Ab1|H2-D1|H2-DMa|H2-DMb1|H2-DMb2|H2-Eb1|H2-K1|H2-Ob|H2-Q10|H2-Q2|H2-Q6|H2-Q7|H2-Q8|H2-T23|Tap1|Tap2|Tapbp | -1.79 | 0.02 |
|  | Intestinal immune network for IgA production | 7.37 | 0.01 | Ccl25\|Cxcl12\|H2-Aa\|H2-Ab1\|H2-DMa\|H2-DMb1\|H2-DMb2\|H2-Eb1\|H2-Ob\|Itgb7\|Pigr\|Tnfrsf17\|Tnfsf13b | -2.01 | 0.01 |
|  | Cell adhesion molecules (CAMs) | 6.25 | 0.01 | CR974466.3\|Cd6\|Cd8a\|Cldn2\|Cldn8\|H2-Aa\|H2-Ab1\|H2-D1\|H2-DMa\|H2-DMb1\|H2-DMb2\|H2-Eb1\|H2-K1\|H2-Ob\|H2-Q10\|H2-Q2\|H2-Q6\|H2-Q7\|H2-Q8\|H2-T23\|Itga8\|Itgb7\|Negr1\|Neo1\|Ptprf | -1.74 | 0.02 |
|  | Toll-like receptor signaling pathway | 2.37 | 0.02 | Ccl5\|Cxcl9\|Ikbke\|Irf7\|Jun\|Mapk9\|Stat1\|Tlr1\|Tlr3 | -1.69 | 0.02 |
| **GO categories overrepresented in upregulated genes** | | | | | | |
| **GOID** | **GO Name** | **Z Score** | **AdjustedP** | **gene symbols** | **AVG-logFC** | **AVG-adj.P.Val** |
| GO:0006954 | inflammatory response | 9.09 | 0.03 | Calca|Ccl2|Ccl3|Ccl4|Cd14|Cd163|Chi3l3|Chi3l4|Cxcl3|Cxcl5|Darc|Fcgr1|Il1b|Il1rn|Il6|Kdm6b|Ltb4r1|Mif|Ptgs2|S100a8|S100a9|Selp|Serpina3n|Slc11a1|Sphk1|Stat3|Thbs1|Tlr6 | 3.33 | 0.04 |
| GO:0042221 | response to chemical stimulus | 9.00 | 0.03 | 1100001G20Rik|Abca1|Abcb11|Abcc1|Acr|Acsl4|Adipor1|Adrbk1|Agps|Akr1c6|Alcam|Aldh1a3|Angptl4|Anxa1|Anxa3|Apbb2|Apob|Apobec1|Areg|Asns|Atp6v0e|Bcl2l1|Calca|Ccl2|Ccl3|Ccl4|Ccl9|Ccng1|Cd14|Clic4|Creb3l2|Creb3l3|Ctgf|Cxcl3|Cxcl5|Cxcr2|Cyp1b1|Cyp2b9|Cyr61|Ddit4|Dusp4|Ecscr|Enah|Ero1l|Etv4|F3|Fbp1|Fcer1g|Fhl2|Fosl1|Fpr1|Gch1|Gclc|Ggh|Gjb4|Gsta1|Gsta2|Hba-a1|Hba-a2|Hmox1|Hoxa1|Hp|Ifnar2|Il1b|Il1r2|Il1rn|Il5ra|Il6|Kcnj8|Krt6a|Lcn2|Lct|Lifr|Lilrb3|Ly6d|Mif|Mmp3|Mmp7|Mmp9|Mt1|Mt2|Nqo1|Nr1i3|Nr4a1|Osmr|Oxsr1|Pappa|Pf4|Pgam2|Phip|Por|Prdx6|Prkacb|Ptgr1|Ptgs2|Ptk2b|Rbp1|S100a8|S100a9|Selp|Serpina3c|Serpina3n|Serpinc1|Slc11a1|Slc23a1|Slc7a11|Sox9|Spp1|Srd5a2|Stat3|Stat6|Steap2|Thbs1|Tlr6|Ubxn4|Upf2|Wapal|Zfp106 | 2.42 | 0.04 |
| GO:0006955 | immune response | 4.04 | 0.03 | Ccl2|Ccl3|Ccl4|Ccl9|Cd14|Clec4d|Cxcl3|Cxcl5|Fcer1g|Fcgr1|Il1b|Il1rn|Il6|Lcn2|Lilrb3|Mif|Pcbp2|Pf4|Ppbp|Procr|Slc11a1|Thbs1|Tlr6 | 3.42 | 0.04 |
| GO:0007155 | cell adhesion | 2.64 | 0.20 | Alcam|Anxa9|Cadm3|Calca|Cass4|Cd33|Cd47|Cd93|Ctgf|Cyp1b1|Cyr61|Dst|Emilin2|Kitl|Lamc2|Lypd3|Lyve1|Mllt4|Msln|Ninj1|Ptk2b|Pvr|Selp|Sox9|Spp1|Thbs1|Tnfrsf12a | 2.04 | 0.04 |
| **GO categories overrepresented in downregulated genes** | | | | | | |
| **GOID** | **GO Name** | **Z Score** | **AdjustedP** | **gene symbols** | **AVG-logFC** | **AVG-adj.P.Val** |
| GO:0019883 | antigen processing and presentation of endogenous antigen | 12.82 | 0.05 | H2-D1|H2-K1|H2-Q7|H2-T23|Tap1|Tap2|Tapbp|Tapbpl | -1.66 | 0.04 |
| GO:0002478 | antigen processing and presentation of exogenous peptide antigen | 8.09 | 0.05 | H2-Aa|H2-Ab1|H2-DMa|H2-DMb1|H2-DMb2|H2-Eb1|H2-K1|Tap2|Tapbp | -2.11 | 0.02 |
| GO:0006955 | immune response | 7.88 | 0.05 | Bst2|C2|CR974466.3|Ccl25|Ccl5|Cd8a|Cxcl12|Cxcl9|Fcgrt|Gm11127|Gm8815|Gzmb|H2-Aa|H2-Ab1|H2-D1|H2-DMa|H2-DMb1|H2-DMb2|H2-Eb1|H2-Gs10|H2-K1|H2-Ob|H2-Q10|H2-Q2|H2-Q6|H2-Q7|H2-Q8|H2-T23|Ifih1|Irf7|Irf8|Irgm1|Nlrx1|Oas1a|Oas1f|Oasl2|Smad6|Spon2|Susd2|Tgtp1|Tgtp2|Tinag|Tlr1|Tlr3|Tnfrsf11a|Tnfsf13b|Zap70 | -1.73 | 0.02 |
| GO:0002504 | antigen processing and presentation of peptide or polysaccharide antigen via MHC class II | 6.48 | 0.05 | H2-Aa|H2-Ab1|H2-DMa|H2-DMb1|H2-DMb2|H2-Eb1|H2-Ob | -2.14 | 0.01 |
| GO:0007155 | cell adhesion | 3.74 | 0.05 | Aoc3|Barx2|Bcl2|Bves|Ccl5|Cd6|Cd96|Cdh11|Cdhr2|Cdhr5|Cdon|Cldn2|Cldn8|Col14a1|Col2a1|Col5a1|Col8a2|Colec10|Cpxm2|Dab1|Efr3a|Fbln7|Gas6|Ihh|Itga11|Itga8|Itgae|Itgb7|Negr1|Neo1|Pgm5|Ptprf|Ptprk|Rgmb|Spon2|Tgfbi|Thra|Tinag|Tmem8|Tnxb | -1.50 | 0.04 |

High scoring functional category groups (KEGG pathways + GO (biological process) categories) in the TNBS-treated ileum are shown in colour as follows: Cytokine-cytokine receptor interaction pathway and inflammatory, immune response category group. Intestinal immune network for IgA production pathway and immune response category group. Antigen processing and presentation pathway and antigen processing and presentation category group. Cell adhesion molecules pathway and cell adhesion category group. Toll-like receptor signaling pathway and immune and inflammatory response category group. ABC transporters pathway and response to chemical stimulus category group. (Note: Although immune, inflammatory response categories are common to more than one category group, they are coloured only once). MAPP Name (KEGG pathway name), Z score (z-score), AdjustedP (adjusted permutation p-value calculated using the Benjamini-Hochberg method)), gene symbols, AVG-logFC (average log_2_FC for each enriched term calculated based on associated differential genes) and AVG-adj.P.Val (average adjusted p-value for each enriched term calculated based on associated differential genes, GOID (Gene ontology ID), GO Name (name of GO category). Threshold criteria for over-representation: z-score>2, at least three genes from the input list in the enriched term, an adjusted permutation p-value≤0.35.
